# Survey and toxigenic abilities of *Aspergillus*, *Fusarium*, and *Alternaria* fungi from wheat and paddy grains in Shanghai, China

**DOI:** 10.3389/fpls.2023.1202738

**Published:** 2023-07-25

**Authors:** Jiajia Meng, Ruijiao Li, Qingwen Huang, Dehua Guo, Kai Fan, Jingya Zhang, Xueting Zhu, Min Wang, Xinyue Chen, Dongxia Nie, Chen Cao, Zhihui Zhao, Zheng Han

**Affiliations:** ^1^ Institute for Agro-food Standards and Testing Technology, Shanghai Academy of Agricultural Sciences, Shanghai, China; ^2^ School of Health Science and Engineering, University of Shanghai for Science and Technology, Shanghai, China; ^3^ Technical Center for Animal Plant and Food Inspection and Quarantine, Shanghai Customs, Shanghai, China

**Keywords:** mycotoxins, toxigenic ability, *Fusarium* spp., *Aspergillus* spp., *Alternaria s*pp

## Abstract

A systematic study was carried out on 638 wheat and paddy grains (including fresh and stored samples) collected in 2021 from Shanghai, China, to identify the major mycobiota and their toxigenic abilities. A total of 349 fungi, namely, 252 *Fusarium*, 53 *Aspergillus*, and 44 *Alternaria*, were characterized by morphological and molecular identification. *Fusarium* and *Aspergillus* were more frequently isolated in paddy with *Fusarium sambucinum* species complex and *Aspergillus* section *flavi* as the predominant species, respectively. The genus *Alternaria* was the most frequently isolated fungal species in wheat. The toxin-producing potentials of the identified fungi were further evaluated *in vitro*. Deoxynevalenol (DON) was produced by 34.5% of *Fusarium* isolates and zearalenone (ZEN) was produced by 47.6% of them, and one isolate also processed the abilities for fumonisin B_1_ (FB_1_), B_2_ (FB_2_), and B_3_ (FB_3_) productions. Aflatoxin B_1_ (AFB_1_), B_2_ (AFB_2_), and G_1_ (AFG_1_) were only generated by *Aspergillus* section *flavi*, with the production rate of 65.5%, 27.6%, and 13.8%, respectively. Alternariol (AOH) was the most prevalent *Alternaria* toxin, which could be produced by 95.5% of the isolates, followed by alternariol monomethyl ether (AME) (72.7%), altenuene (ALT) (52.3%), tenuazonic acid (TeA) (45.5%), tentoxin (TEN) (29.5%), and altenusin (ALS) (4.5%). A combinational analysis of mycobiota and toxigenic ability allowed us to provide comprehensive information about the production mechanisms of mycotoxins in wheat and paddy in a specific geographic area, and will be helpful for developing efficient prevention and control programs.

## 1 Introduction

Paddy (*Oryza sativa* L.) and wheat (*Triticum aestivum* L.), widely cultured crops in the world, are considered as the most important staple foods in China (Hou et al., 2015). Shanghai, the center of China’s economy and trade (Wang and Zhang, 2005), imports most of its consumed wheat and paddy grains (approximately 80.0%) from other cities, such as Anhui, Shandong, and Heilongjiang. The quality and safety of the stored grains are essential, and any deterioration may lead to a significant impact on consumer and animal health.

In Shanghai, the typical subtropical monsoon climate provides favorable conditions for fungal infections of grains (Chen et al., 2008; Qiu et al., 2014). With suitable temperature and surface moisture, fungal spores present on the kernels can germinate and grow, and then destroy the kernels, leading to yield loss and quality reduction (Lacey and Magan, 1991; Kosiak et al., 2004). Some fungal species can also produce a range of mycotoxins (Luo et al., 2021), which are chemically or heat stable, and difficult to be degraded, leading to various adverse health effects including teratogenicity, carcinogenicity, mutagenicity, immunotoxicity, or neurotoxicity (Haque et al., 2020; Suman, 2021). Mycotoxin contamination is considered to be one of the most serious food safety problems in the world (Ali et al., 2022).

In China, many studies have reported the serious contaminations of mycotoxins in wheat and paddy grains, mainly focusing on *Fusarium* toxins [e.g., deoxynevalenol (DON), zearalenone (ZEN), fumonisin B_1_ (FB_1_), B_2_ (FB_2_), and B_3_ (FB_3_)] (Han et al., 2014; Qiu et al., 2019; Yan et al., 2020), *Aspergillus* toxins [e.g., aflatoxin B_1_ (AFB_1_), B_2_ (AFB_2_), G_1_ (AFG_1_), and G_2_ (AFG_2_)] (Sun et al., 2011; Li et al., 2014), and *Alternaria* toxins [e.g., alternariol (AOH), alternariol monomethyl ether (AME), tenuazonic acid (TeA), tentoxin (TEN), altenuene (ALT), and altenusin (ALS)] (Li et al., 2001; Xu et al., 2016; Jiang et al., 2021). A 3-year (2010–2012) survey, conducted in Jiangsu province, China, showed that DON was the most important mycotoxin, which was found in 74.4% of wheat samples at levels ranging from 14.5 to 41,157.1 μg/kg (mean, 488.0 μg/kg), while ZEN was detected in 12.8% of samples at levels ranging from 10.1 to 3,048.9 μg/kg (mean, 73.0 μg/kg) (Ji et al., 2014). Owing to the widespread occurrence and high toxicities, comprehensive information on the production mechanisms of typical mycotoxins have become a critical issue.

In general, production of particular mycotoxins by fungi primarily depends on the fungal species. As reported, DON and ZEN are mainly produced by *F. graminearum* and *F. culmorum* (Yang et al., 2018; Ekwomadu et al., 2021); AFB_1_, AFB_2_, AFG_1_, and AFG_2_ are mainly produced by *A. flavus* and *A. parasiticus* (Tsai and Yu, 1999; Diaz et al., 2009); *Alternaria* toxins are mainly produced by *A. alternata*, *A. padwickii*, etc. (Ntasiou et al., 2015; Turzhanova et al., 2020). Certain mycotoxins in grains could also be produced by others fungal species. Fumonisins are mainly produced by species of *Fusarium fujikuroi* complex such as *F. verticillioides*, *F. proliferatum*, and *F. fujikuroi*, but they could also be produced by *Aspergillus* spp. (Frisvad et al., 2007). The toxigenic abilities of the strains belonging to the same species vary in types and concentrations of the produced mycotoxins. The same fungi might even produce different mycotoxins under different environmental conditions. *A. alternata* was known to produce *Alternaria* toxins, but it could also produce fumonisins (Chen et al., 1993; Abbas and Riley, 1996; Mirocha et al., 1996). To date, although mycotoxin contamination in wheat and paddy grains in Shanghai, China has been reported (Xing et al., 1997; Fan et al., 2021; Huang et al., 2022), little was known about the occurrence of the main toxigenic fungi and their abilities for mycotoxin production.

Based on these considerations, the aims of this work were to (1) investigate the presence of fungal microorganisms, with particular attention to toxigenic species, in 638 wheat and paddy samples collected from Shanghai, China in 2021; and (2) evaluate the mycotoxin-producing potentials of the main isolates including *Aspergillus* spp., *Fusarium* spp., and *Alternaria* spp.

## 2 Materials and methods

### 2.1 Chemicals and reagents

The mycotoxin standards (purity>98%) of AFB_1_, AFB_2_, AFG_1_, AFG_2_, DON, ZEN, AOH, AME, TeA, TEN, ALT, ALS, FB_1_, FB_2_, and FB_3_ (**Supplementary Figure 1**) were purchased from Qingdao pribolab (Qingdao, China). All standards were dissolved in acetonitrile to prepare 1.0 mg/ml of stock solutions and stored at −20 ± 2°C. Water was purified by a Milli-Q system (Millipore, Brussels, Belgium).

Methanol, acetonitrile, formic acid, and ammonium acetate (HPLC grade) were purchased from Merck (Darmstadt, Germany). Sodium chloride (NaCl, analytical grade) and anhydrous magnesium sulfate (MgSO_4_, analytical grade) were supplied by ANPEL (Shanghai, China).

### 2.2 Grain samples

A total of 638 grain samples, namely, 365 wheat (56 fresh wheat and 309 stored wheat) and 273 paddy (119 fresh paddy and 154 stored paddy), were collected from Shanghai Pujiang Warehousing Co., Ltd. (Shanghai, China) in 2021. The fresh grains were the samples freshly collected from different parts of China (**Supplementary Figure 2**) and shipped to Shanghai for storage (**Table 1**). The stored grains were the samples stored in the barns of Shanghai for 2–5 years with good ventilation and controllable temperature (10–20°C) and humidity (50%–60%) conditions. All collected samples (each approximately 500 g) were stored in pre-sterilized polyethylene bags at 4.0 ± 0.5°C until analysis.

**Table 1:** Information of the collected grain samples.

Type	Origin	Storage Time (years)	Wheat	Paddy
Fresh grains	Jiangsu	0	35	41
	Shanghai	0	0	76
	Shandong	0	19	0
	Anhui	0	2	2
Store grains	Jiangsu	2	178	0
	Shanghai	3	0	60
	Shandong	5	131	0
	Heilongjiang	4	0	94

### 2.3 Isolation and identification of fungal strains

Seeds were soaked in 75% ethanol for 2 min, and then were rinsed three times by sterile water. The surface moisture of the seeds was wiped with sterilized absorbent paper. The seeds were then placed on the surface of potato dextrose agar (PDA) in petri dishes (90 mm diameter, 8 kernels/plate) and incubated at 28 ± 2°C for 4 days. The fungal strains were purified by subculture of single conidia (Dong et al., 2021) and stored as spores in 30% glycerol at −80 ± 2°C.

The isolated fungi were firstly identified by the morphological observations according to the previous studies (Reddy et al., 2010; Nagaraja et al., 2016; Tralamazza et al., 2018) and then were validated by PCR analysis (Munitz et al., 2014). All fungal strains were inoculated on PDA and cultured for 7 days at 28 ± 2°C in the dark. DNA was extracted from fungal strains according to the CTAB protocol and dissolved in 50 µl of TE (pH 8.0, 10 mM Tris and 1 mM EDTA) (Brandfass and Karlovsky, 2008). The universal primers ITS1 (5-TCCGTAGGTGAACCTGCGG-3) and ITS4 (5-TCCTCCGCTTATTGATATGC-3) were selected and the total volume of the PCR amplification was 50 μl. The PCR reaction conditions were as follows: 95°C for 5 min, followed by 35 cycles at 95°C for 30 s, 58°C for 30 s, 72°C for 1 min, and finally 72°C extension for 7 min. PCR products were purified by the AxyPrep DNA gel recovery kit, and sequenced with ABI 3730XL Analyzer (Applied Biosystems) (Sunagawa et al., 2021). The ITS sequences were compared with the sequences in the National Center for Biotechnology Information (NCBI) GenBank database by the Basic Local Alignment Search Tool (BLAST) (**Supplementary Table 1**). The isolates were identified with the sequences similarity in the range of 99%–100%.

### 2.4 Mycotoxin production by the isolated fungal strains

The toxigenic abilities of the fungal strains, which were confirmed as *Fusarium*, *Aspergillus*, or *Alternaria*, were evaluated in PDA. Samples were prepared following the method described previously (Fan et al., 2021). Briefly, the isolated fungal strains were cultured on PDA (9 mm diameter agar disc) at 28 ± 2°C for 7 days in quintuplicate (*n* = 5). The medium was dried at 50 ± 2°C (Shi et al., 2016), and the weight of medium was recorded for calculation of the mycotoxin production. Then, it was transferred into a 50-ml centrifuge tube and extracted with 10 ml of acetonitrile/water/formic acid (84/15/1, v/v/v) by shaking for 30 min and ultrasonicating for 40 min. After centrifugation at 4,000 *g* for 10 min, the supernatant was collected and evaporated under a soft stream of nitrogen gas at 45°C. The residues were redissolved in 1 ml of acetonitrile/water containing 5 mmol/L ammonium acetate (20/80, v/v). Finally, the solution was passed through a 0.22-μm filter membrane prior to ultrahigh-performance liquid chromatography-tandem mass spectrometry (UPLC-MS/MS) analysis.

### 2.5 UPLC-MS/MS analysis

UPLC analysis was performed on a Waters Acquity UPLC system (Waters, Milford, MA, USA). Separation was achieved on a Waters XBridge® BEH-C_18_ XP column (130 Å, 2.5 µm, 3.0 × 100 mm, PN: 186006035) at 40°C. The mobile phase consisted of (A) acetonitrile and (B) water containing 5 mmol/L ammonium acetate, and a linear gradient elution program was applied as follows: initial, 10% A; 1 min, 10% A; 3 min, 70% A; 5 min, 90% A; 6 min, 90% A; 6.1 min, 10%; 8 min, 10% A. The mobile phase flow rate was 0.4 ml/min.

The separated compounds were analyzed by a Waters XEVO TQMS mass spectrometer (Waters, Milford, MA, USA) with an electrospray ionization source operated in negative mode (ESI^−^) for ZEN and ALS, and in positive mode (ESI^+^) for the other mycotoxins. Multiple reaction monitoring (MRM) mode was established as shown in **Supplementary Table 2**. The source parameters are set as follows: capillary voltage of 2.5 kV for ESI^+^ and 1.5 kV for ESI^−^, ion source temperature of 150°C, and desolvation temperature of 500°C. The gas flow rates were 7.0 bar for nebulizing gas and 1,000 L/h for desolvation gas, respectively. TargetLynx XS software was used to process the data (Waters Corporation, Milford, MA, USA).

### 2.6 Statistics

Tables were plotted using Microsoft Office Excel 2019 (Microsoft Corp., Redmond, WA, USA). Mycotoxin analysis was performed using TargetLynx XS software (Waters Corporation, Milford, MA, USA). The statistical analysis was performed using IBM SPSS Statistics soft version 26.0 (SPSS Inc., Chicago, IL, USA). The effect of fungal species on the production of mycotoxins was analyzed by a Chi-square test. Meanwhile, the effect of wheat/paddy and fresh/stored on the production of mycotoxins by fungi was analyzed by one-way ANOVA based on *t*-test, differences with *p* value ≤ 0.05 were considered significant. The DNA sequences were edited and aligned by BLAST at NCBI (http://www.ncbi.nlm.nih.gov/).

## 3 Results

### 3.1 Occurrence of fungal species from 638 wheat and paddy samples in Shanghai, China

A total of 349 fungal isolates (see **Supplementary Table 1** for details) were obtained from 638 wheat and paddy samples in Shanghai, China (**Table 2**). The number of isolates (298) from paddy were much more than those (51) from wheat. The most prevalent genus was *Fusarium* with 252 isolates recovered. Among these, 242 isolates were isolated from fresh paddy grains. According to the morphological study and ITS sequences, *Fusarium* species were further characterized as members of the certain species complex (SC). *Fusarium sambucinum* SC were identified to be the predominant fungi. It is worth noting that 45.4% of the samples were infected with more than one *Fusarium* strain.

**Table 2:** Number of isolates from different grains.

Fungi	Wheat (*n* = 365)		Paddy (*n* = 273)		Total Number	Proportion (%)
	Fresh (*n* = 56)	Stored (*n* = 309)	Fresh (*n* = 119)	Stored (*n* = 154)		
*Fusarium sambucinum* SC	3	1	141	0	145	41.5
*Fusarium fujikuroi* SC	0	0	4	3	7	2.0
Other *Fusarium* spp.	1	2	97	0	100	28.7
*Aspergillus* section *flavi*	0	9	0	20	29	8.3
*Aspergillus fumigatus*	0	0	0	12	12	3.4
Other *Aspergillus* spp.	0	4	0	8	12	3.4
*Alternaria* spp.	19	12	13	0	44	12.6
Total	23	28	255	43	349	100

n denotes the number of samples.

*Aspergillus* spp. (53) were also isolated but at a relatively lower frequency (15.2%) compared to *Fusarium* spp. (72.2%). All *Aspergillus* isolates were identified from stored grains with more strains (40) from paddy than that (13) from wheat. *Aspergillus* section *flavi* and *A. fumigatus* were the dominant species. Conversely, a total of 44 *Alternaria* spp. were isolated, most of which were from fresh grains (32), and no strains were found in stored paddy grains.

### 3.2 Toxigenic abilities of the main isolates

The toxin-producing potentials of the 349 isolates belonging to *Fusarium* spp., *Aspergillus* spp., and *Alternaria* spp. were evaluated (**Supplementary Table 3**). All the isolates were cultured in PDA for 7 days at 28 ± 2°C. As shown in **Table 3**, among 252 *Fusarium* strains, 87 (34.5%) isolates could produce DON, and 120 (47.6%) could produce ZEN. One out of three *Fusarium* isolates from stored wheat could produce DON, with a level of 44.2 mg/kg, much higher than those from fresh wheat grains. As potential producers of fumonisins, all the *Fusarium* isolates were further analyzed. Only one *Fusarium fujikuroi* SC stain isolated from fresh paddy was found to produce fumonisins with the concentrations of 128.1 mg/kg for FB_1_, 39.2 mg/kg for FB_2_, and 38.9 mg/kg for FB_3_, respectively.

**Table 3:** *Fusarium* toxin-producing potentials of *Fusarium* species isolated from different grains in Shanghai, China.

Grains (Fusarium isolates)	Type	DON		ZEN	
		No. of positive samples of isolates	Average level (range) (mg/kg)	No. of positive samples of isolates	Average level (range) (mg/kg)
Wheat (*n* = 7)	Fresh (*n* = 4)	3	0.3 (0.2–0.5)	3	6.1 (0.3–9.3)
	Stored (*n* = 3)	1	44.2 (44.2)	1	3.8 (3.8)
Paddy (*n* = 245)	Fresh (*n* = 242)	83	2.0 (0.1–18.4)	116	28.82 (0.01–893.3)
	Stored (*n* = 3)	0	/	0	/

n denotes the number of *Fusarium* isolates.

The abilities of *Aspergillus* isolates to produce AFB_1_, AFB_2_, AFG_1_, and AFG_2_ were evaluated (**Table 4**). Among 53 *Aspergillus* strains, only 29 *Aspergillus* section *flavi* could produce AFB_S_, from which, 19 (35.8%) produced AFB_1_, 8 (15.1%) produced AFB_2_, 4 (7.5%) produced AFG_1_, and no one could produce AFG_2_. The highest levels of AFB_1_, AFB_2_, and AFG_1_ were 155.5 mg/kg, 19.0 mg/kg, and 0.6 mg/kg, respectively, which were produced by the same *Aspergillus* section *flavi* isolate.

**Table 4:** *Aspergillus* toxin-producing potentials of *Aspergillus* isolates from different grains in Shanghai, China.

Grains (*Aspergillus* isolates)	Type	AFB_1_		AFB_2_		AFG_1_		AFG_2_	
		No. ^a^	Average level (range ^b^) (mg/kg)	No.	Average level (range) (mg/kg)	No.	Average level (range) (mg/kg)	No.	Average level (range) (mg/kg)
Wheat (*n* = 13)	Fresh (*n* = 0)	0	/	0	/	0	/	0	/
	Stored (*n* = 13)	2	1.78 (0.02–3.5)	1	0.2 (0.2)	0	/	0	/
Paddy (*n* = 40)	Fresh (*n* = 0)	0	/	0	/	0	/	0	/
	Stored (*n* = 40)	17	24.0 (0.1–155.5)	7	5.6 (0.01–19.0)	4	0.2 (0.1–0.6)	0	/

n denotes the number of *Aspergillus* isolates. ^a^ means the number of positive samples of isolates.

The abilities of *Alternaria* isolates (44) were also assayed for their productions of six *Alternaria* toxins, including AOH, AME, TeA, TEN, ALT, and ALS (**Table 5**). A total of 95.5% *Alternaria* isolates could produce at least one *Alternaria* toxin. AOH was the most prevalent *Alternaria* toxin, which could be produced by 95.5% of the isolates, followed by AME (72.7%), ALT (52.3%), TeA (45.5%), and TEN (29.5%). Only two *Alternaria* isolates produced ALS, both of which were isolated from wheat grains.

**Table 5:** *Alternaria* toxin-producing potentials of *Alternaria* isolates from different grains in Shanghai, China.

Grains *(Alternaria* isolates)	Type	AOH		AME		TeA		TEN		ALT		ALS	
		No.^a^	Average level (range) (mg/kg)	No.	Average level (range) (mg/kg)	No.	Average level (range) (mg/kg)	No.	Average level (range) (mg/kg)	No.	Average level (range) (mg/kg)	No.	Average level (range) (mg/kg)
Wheat (*n* = 31)	Fresh (*n* = 19)	18	322.6 (2.7–2,328.1)	15	91.2 (0.5–770.0)	10	240.2 (2.6–1,410.0)	4	14.6 (0.5–45.2)	9	216.8 (16.1–1,395.2)	1	275.8 (275.8)
	Stored (*n* = 12)	12	258.3 (3.7–1,654.0)	8	321.5 (0.2–2,261.0)	7	64.3 (2.7–191.5)	5	0.6 (0.1–1.4)	7	81.1 (14.7–205.2)	1	424.5 (424.5)
Paddy (*n* = 13)	Fresh (*n* = 13)	12	233.4 (3.1–1,441.1)	9	47.2 (11.0–175.5)	3	144.1 (11.1–245.1)	4	2.4 (0.1–5.6)	7	80.6 (3.1–278.1)	0	/
	Stored (*n* = 0)	0	/	0	/	0	/	0	/	0	/	0	/

n denotes the number of *Alternaria* isolates. ^a^ means the number of positive samples of isolates.

## 4 Discussion

**Figure 1:**
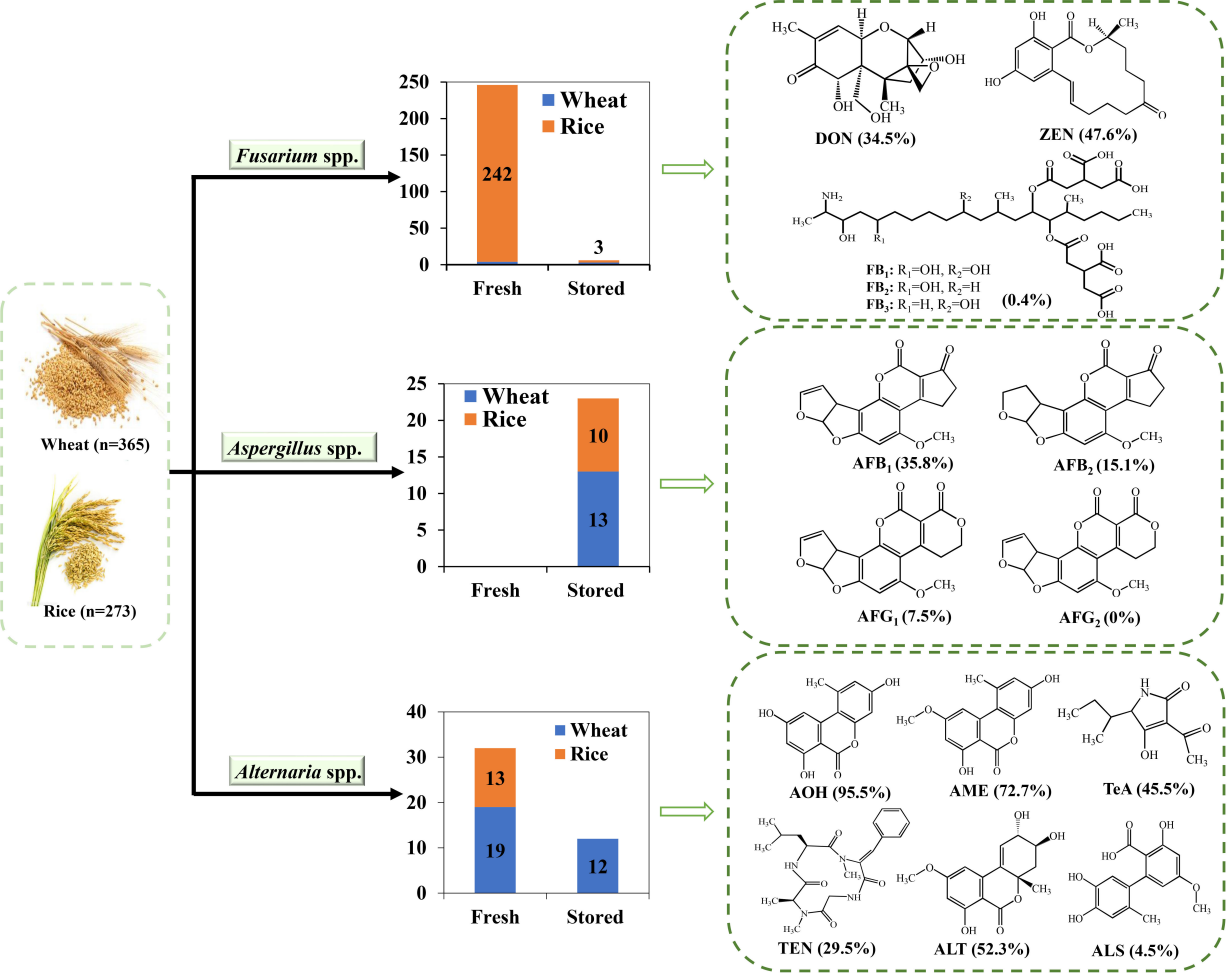
The distributions of *Fusarium* spp., *Aspergillus* spp., and *Alternaria* spp. in different grains in Shanghai, China, and their toxigenic abilities.

Shanghai is the economic and financial center of China rather than a major agricultural city and most of wheat and paddy consumed in this city were supplied by other areas of China. Mycotoxins, a series of secondary metabolites produced by various mold species in grains, especially wheat and paddy during storage, have become important impactors on human and animal health. Consequently, the presence and toxigenic abilities of the harmful fungi in the stored grains are of great concern. In this study, a total of 638 wheat and paddy grains were collected from Shanghai, China in 2021, and the presence of probable toxigenic fungi including *Fusarium* spp., *Aspergillus* spp., and *Alternaria* spp. And their toxin-producing potentials were thoroughly investigated (**Figure 1**). To the best of our knowledge, this is the first survey conducted on wheat and rice grains (including fresh and stored samples) consumed in Shanghai, China that took into consideration both contaminating fungi and their toxigenic abilities. However, the morphological study in combination with ITS region analysis was insufficient to distinguish the specific sections but only provided genera information. Further molecular identification is required to accurately identify the species of *Fusarium*, *Aspergillus*, and *Alternaria*.

The differences of occurrence and toxigenic abilities of *Fusarium*, *Aspergillus*, and *Alternaria* species from wheat and paddy grains in Shanghai, China, were compared. The results showed that *Fusarium* spp. were the predominant species in fresh grains, and *Aspergillus* spp. were predominant in stored grains. The serious contaminations of *Fusarium* spp. in fresh samples might be due to the colonization of the fungi in the field, poor management, or damp conditions during the harvesting phase and transportation (Magan and Aldred, 2007; Magan et al., 2010). *Aspergillus* spp. is normally considered as the fungi developed in stored commodities and widely discovered in stored grains (Medina et al., 2006; Riba et al., 2010; Alkuwari et al., 2022; Tournas and Niazi, 2018; Zhao et al., 2020). All *Aspergillus* strains were isolated from stored grains, and 47.5% could produce AFBs. Interestingly, all the isolated *Alternaria* spp. were capable of producing at least one *Alternaria* toxin, whether in fresh or in stored grains.

Among the fungal communities recovered, *Fusarium* spp. were the dominant fungi in paddy grains. Most of the isolated *Fusarium* spp. produced DON and ZEN, and a large number of the *Fusarium* isolates could even co-produce DON and ZEN. The co-contaminations of DON and ZEN in wheat and paddy grains have been widely reported in literatures (Qiu and Shi, 2014; Dong et al., 2020; Fan et al., 2021), proving these organisms to be the common spoilers of grains. On the other hand, the co-occurrence of different *Fusarium* toxins might cause joint toxicities to humans and animals, which should be paid more attention in the future.

With regard to *Aspergillus* spp., the incidence was lower than that indicated by other authors, who collected the samples mainly from diseased grains (Chehri et al., 2015). As the predominant *Aspergillus* spp., *Aspergillus* section *flavi* might be associated with warmer geographical regions, similar to the previous studies conducted in Turkey, Iran, Australia, and Argentina (Berghofer et al., 2003; Vaamonde et al., 2003; Baydar et al., 2005; Chehri et al., 2015). Among 53 *Aspergillus* isolates, only *Aspergillus* section *flavi* could produce AFBs. The same results were discovered by Riba et al. in Algerian wheat, in which *A. flavus* was the only aflatoxigenic fungus among all the *Aspergillus* isolates (Riba et al., 2010). Different toxigenic abilities have also been described, in that some fungi could produce four AFBs (AFB_1_, AFB_2_, AFG_1_, and AFG_2_), while others only produced either three or two AFBs (Saleemi et al., 2010).

In recent years, *Alternaria* spp. have been pointed out as important contaminants in grains, especially in some regions with warm and humid climates (Li and Yoshizawa, 2000; Li et al., 2001). The incidence of *Alternaria* spp. in the current study was lower than that in Anhui province (100.0%), where the temperature and humidity were higher (Xu et al., 2016). In comparison to paddy, *Alternaria* spp. were more frequently found in wheat samples, which was in good agreement with the previous studies in China (Li et al., 2001; Xu et al., 2016). Almost all the isolates (95.5%) could produce at least one *Alternaria* toxin, among which AOH, AME, TeA, and TEN were the most frequently found, similar to the surveys from Germany (Muller and Korn, 2013), Canada (Scott et al., 2012), and Russia (Orina et al., 2022). Potential health risks related to the contaminations of *Alternaria* toxins in grains were thus proposed.

## 5 Conclusions

In the present study, the occurrence and toxigenic abilities of *Fusarium*, *Aspergillus*, and *Alternaria* species from wheat and paddy grains in Shanghai, China, were evaluated. *Fusarium* spp. were the main species in fresh grains, and *Aspergillus* spp. were predominant in stored grains. Toxin-producing potentials were different depending on the types and sources of the isolated fungi, from which a series of typical mycotoxins including DON, ZEN, AFBs, FBs, and *Alternaria* toxins could be generated. Co-productions of different secondary metabolites by toxigenic fungi could lead to co-contaminations of multiple mycotoxins, posing potentially additional health risks to humans and animals.

## Data availability statement

The original contributions presented in the study are included in the article/**Supplementary Materials**, further inquiries can be directed to the corresponding author/s.

## Author contributions

JM and ZH performed the entire project together, collected the data, performed the data analysis, and wrote the manuscript. RL, QH, JZ, XZ, XC, and CC processed the samples. KF, MW, and DG contributed to the data analysis. DN, ZZ, and ZH supervised the project. All authors contributed to the article and approved the submitted version.
