# Supplementary material for: Survey and toxigenic abilities of Aspergillus, Fusarium, and Alternaria fungi from wheat and paddy grains in Shanghai, China
Source: Front Plant Sci. 2023 Jul 25;14:1202738. doi: 10.3389/fpls.2023.1202738 (PMC10407302; doi:10.3389/fpls.2023.1202738)
Supplement: Supplementary file 2 [file DataSheet_1.docx]

Supplementary Material

Survey and Toxigenic Abilities of *Aspergillus*, *Fusarium* and *Alternaria* Fungi from Wheat and Paddy Grains in Shanghai, China

**Jiajia Meng ^1^, Ruijiao Li ^2^, Qingwen Huang ^1^, Dehua Guo ^3^, Kai Fan ^1^, Jingya Zhang ^1^, Xueting Zhu ^1^, Min Wang ^3^, Xinyue Chen ^1^, Dongxia Nie ^1^, Chen Cao ^3^, Zhihui Zhao ^1^ and Zheng Han ^1,2*^**

*** Correspondence:** Zheng Han: [hanzheng@saas.sh.cn](mailto:hanzheng@saas.sh.cn)

# Supplementary Figures and Tables

## Supplementary Figures

**Supplementary Figure 1.** Chemical structures of deoxynevalenol (DON), zearalenone (ZEN), fumonisin B_1_ (FB_1_), fumonisin B_2_ (FB_2_), fumonisin B_3_ (FB_3_), aflatoxin B_1_ (AFB_1_), aflatoxin B_2_ (AFB_2_), aflatoxin G_1_ (AFG_1_), aflatoxin G_2_ (AFG_2_), alternariol (AOH), alternariol monomethyl ether (AME), altenuene (ALT), altenusin (ALS) tenuazonic acid (TeA), and tentoxin (TEN).

**Supplementary Figure 2.** Map of China showing the locations of samples source.

## Supplementary Tables

**Supplementary Table 1**. Information of the fungi isolated from wheat and paddy grains.

| **Strain** | **Species** | **Reference species** | **Reference strain** | **Similarity** | **GenBank accession no.** |
| --- | --- | --- | --- | --- | --- |
| 1 | *Alternaria* spp. | *Alternaria alternata* | OP594615 | 100.00% | OQ421550 |
| 2 | *Alternaria* spp. | *Alternaria alternata* | KM458821 | 100.00% | OQ421551 |
| 3 | *Alternaria* spp. | *Alternaria alternata* | KM458821 | 100.00% | OQ421552 |
| 4 | *Alternaria* spp. | *Alternaria alternata* | KM458821 | 100.00% | OQ421553 |
| 5 | *Alternaria* spp. | *Alternaria alternata* | MK332247 | 100.00% | OQ421554 |
| 6 | *Alternaria* spp. | *Alternaria alternata* | OQ001035 | 99.65% | OQ421555 |
| 7 | *Alternaria* spp. | *Alternaria alternata* | OP594615 | 100.00% | OQ421556 |
| 8 | *Alternaria* spp. | *Alternaria alternata* | MK332247 | 100.00% | OQ421557 |
| 9 | *Alternaria* spp. | *Alternaria alternata* | MN173818 | 99.81% | OQ421558 |
| 10 | *Alternaria* spp. | *Alternaria alternata* | MT453271 | 100.00% | OQ421559 |
| 11 | *Alternaria* spp. | *Alternaria alternata* | MH521173 | 100.00% | OQ421560 |
| 12 | *Alternaria* spp. | *Alternaria alternata* | MT453271 | 100.00% | OQ421561 |
| 13 | *Alternaria* spp. | *Alternaria alternata* | MT646481 | 99.65% | OQ421562 |
| 14 | *Alternaria* spp. | *Alternaria alternata* | MK332247 | 100.00% | OQ421563 |
| 15 | *Alternaria* spp. | *Alternaria alternata* | KT192411 | 100.00% | OQ421564 |
| 16 | *Alternaria* spp. | *Alternaria alternata* | MW509979 | 100.00% | OQ421565 |
| 17 | *Alternaria* spp. | *Alternaria alternata* | OQ001035 | 99.65% | OQ421566 |
| 18 | *Alternaria* spp. | *Alternaria alternata* | OP850817 | 100.00% | OQ421567 |
| 19 | *Alternaria* spp. | *Alternaria alternata* | MK972905 | 100.00% | OQ421568 |
| 20 | *Alternaria* spp. | *Alternaria alternata* | MN589682 | 100.00% | OQ421569 |
| 21 | *Alternaria* spp. | *Alternaria alternata* | OK093405 | 100.00% | OQ421570 |
| 22 | *Alternaria* spp. | *Alternaria alternata* | MK972909 | 100.00% | OQ421571 |
| 23 | *Alternaria* spp. | *Alternaria alternata* | MK972909 | 100.00% | OQ421572 |
| 24 | *Alternaria* spp. | *Alternaria alternata* | MK910060 | 100.00% | OQ421573 |
| 25 | *Alternaria* spp. | *Alternaria alternata* | MT420645 | 100.00% | OQ421574 |
| 26 | *Alternaria* spp. | *Alternaria alternata* | ON711950 | 100.00% | OQ421575 |
| 27 | *Alternaria* spp. | *Alternaria alternata* | OP594615 | 100.00% | OQ421576 |
| 28 | *Alternaria* spp. | *Alternaria alternata* | KM100451 | 99.83% | OQ421577 |
| 29 | *Alternaria* spp. | *Alternaria alternata* | OP594615 | 99.83% | OQ421578 |
| 30 | *Alternaria* spp. | *Alternaria alternata* | MK370641 | 99.83% | OQ421579 |
| 31 | *Alternaria* spp. | *Alternaria alternata* | MK332247 | 100.00% | OQ421580 |
| 32 | *Alternaria* spp. | *Alternaria brassicae* | JN108911 | 100.00% | OQ421540 |
| 33 | *Alternaria* spp. | *Alternaria brassicae* | KU204772 | 99.83% | OQ421541 |
| 34 | *Alternaria* spp. | *Alternaria brassicae* | KU204772 | 99.83% | OQ421542 |
| 35 | *Alternaria* spp. | *Alternaria brassicae* | KU204773 | 99.65% | OQ438160 |
| 36 | *Alternaria* spp. | *Alternaria brassicae* | MT635275 | 99.64% | OQ438161 |
| 37 | *Alternaria* spp. | *Alternaria brassicae* | JF439440 | 99.30% | OQ421543 |
| 38 | *Alternaria* spp. | *Alternaria padwickii* | OM899918 | 99.80% | OQ421536 |
| 39 | *Alternaria* spp. | *Alternaria padwickii* | GU373645 | 100.00% | OQ421537 |
| 40 | *Alternaria* spp. | *Alternaria padwickii* | GU373650 | 100.00% | OQ421538 |
| 41 | *Alternaria* spp. | *Alternaria porri* | KT803064 | 100.00% | OQ421532 |
| 42 | *Alternaria* spp. | *Alternaria tenuissima* | MN593339 | 99.66% | OQ438146 |
| 43 | *Alternaria* spp. | *Alternaria tenuissima* | OQ001058 | 99.02% | OQ438163 |
| 44 | *Alternaria* spp. | *Alternaria tenuissima* | MN593335 | 99.83% | OQ438147 |
| 45 | *Fusarium sambucinum* SC | *Fusarium graminearum* | HQ832817 | 100.00% | OQ438079 |
| 46 | *Fusarium sambucinum* SC | *Fusarium graminearum* | OL364745 | 99.82% | OQ438080 |
| 47 | *Fusarium sambucinum* SC | *Fusarium graminearum* | OL364745 | 100.00% | OQ438081 |
| 48 | *Fusarium sambucinum* SC | *Fusarium graminearum* | MG274308 | 100.00% | OQ438082 |
| 49 | *Fusarium sambucinum* SC | *Fusarium graminearum* | OL364745 | 99.65% | OQ438083 |
| 50 | *Fusarium sambucinum* SC | *Fusarium graminearum* | KY910868 | 100.00% | OQ438084 |
| 51 | *Fusarium sambucinum* SC | *Fusarium graminearum* | MT185460 | 100.00% | OQ438085 |
| 52 | *Fusarium sambucinum* SC | *Fusarium graminearum* | KR047057 | 99.82% | OQ438086 |
| 53 | *Fusarium sambucinum* SC | *Fusarium graminearum* | MT185460 | 100.00% | OQ438087 |
| 54 | *Fusarium sambucinum* SC | *Fusarium graminearum* | KY910868 | 100.00% | OQ438088 |
| 55 | *Fusarium sambucinum* SC | *Fusarium graminearum* | MK079918 | 100.00% | OQ438089 |
| 56 | *Fusarium sambucinum* SC | *Fusarium graminearum* | LT222056 | 100.00% | OQ438090 |
| 57 | *Fusarium sambucinum* SC | *Fusarium graminearum* | MT185460 | 100.00% | OQ438091 |
| 58 | *Fusarium sambucinum* SC | *Fusarium graminearum* | LT222056 | 100.00% | OQ438092 |
| 59 | *Fusarium sambucinum* SC | *Fusarium graminearum* | LT222056 | 100.00% | OQ438093 |
| 60 | *Fusarium sambucinum* SC | *Fusarium graminearum* | KY466811 | 99.80% | OQ438094 |
| 61 | *Fusarium sambucinum* SC | *Fusarium graminearum* | MK079918 | 100.00% | OQ438095 |
| 62 | *Fusarium sambucinum* SC | *Fusarium graminearum* | MT185460 | 100.00% | OQ438096 |
| 63 | *Fusarium sambucinum* SC | *Fusarium graminearum* | KY910868 | 99.45% | OQ438097 |
| 64 | *Fusarium sambucinum* SC | *Fusarium graminearum* | LT222056 | 100.00% | OQ438098 |
| 65 | *Fusarium sambucinum* SC | *Fusarium graminearum* | MT185460 | 100.00% | OQ438099 |
| 66 | *Fusarium sambucinum* SC | *Fusarium graminearum* | MT185456 | 100.00% | OQ438100 |
| 67 | *Fusarium sambucinum* SC | *Fusarium graminearum* | LT222056 | 100.00% | OQ438101 |
| 68 | *Fusarium sambucinum* SC | *Fusarium graminearum* | ON024860 | 100.00% | OQ438102 |
| 69 | *Fusarium sambucinum* SC | *Fusarium graminearum* | MT185460 | 99.82% | OQ438103 |
| 70 | *Fusarium sambucinum* SC | *Fusarium graminearum* | MT185460 | 99.82% | OQ438104 |
| 71 | *Fusarium sambucinum* SC | *Fusarium graminearum* | KY910868 | 100.00% | OQ438105 |
| 72 | *Fusarium sambucinum* SC | *Fusarium graminearum* | LT222056 | 99.82% | OQ438106 |
| 73 | *Fusarium sambucinum* SC | *Fusarium graminearum* | MG274308 | 100.00% | OQ438107 |
| 74 | *Fusarium sambucinum* SC | *Fusarium graminearum* | LT222056 | 99.82% | OQ438108 |
| 75 | *Fusarium sambucinum* SC | *Fusarium graminearum* | LT222056 | 100.00% | OQ438109 |
| 76 | *Fusarium sambucinum* SC | *Fusarium graminearum* | ON054308 | 100.00% | OQ438110 |
| 77 | *Fusarium sambucinum* SC | *Fusarium graminearum* | OL364745 | 99.82% | OQ438111 |
| 78 | *Fusarium sambucinum* SC | *Fusarium graminearum* | MT185460 | 99.64% | OQ438112 |
| 79 | *Fusarium sambucinum* SC | *Fusarium graminearum* | MG274308 | 100.00% | OQ438113 |
| 80 | *Fusarium sambucinum* SC | *Fusarium graminearum* | MH591453 | 99.65% | OQ438290 |
| 81 | *Fusarium sambucinum* SC | *Fusarium graminearum* | ON054308 | 99.82% | OQ438114 |
| 82 | *Fusarium sambucinum* SC | *Fusarium graminearum* | OL364745 | 100.00% | OQ438115 |
| 83 | *Fusarium sambucinum* SC | *Fusarium graminearum* | OL364745 | 100.00% | OQ438116 |
| 84 | *Fusarium sambucinum* SC | *Fusarium graminearum* | MK460853 | 99.82% | OQ438117 |
| 85 | *Fusarium sambucinum* SC | *Fusarium graminearum* | MT185460 | 100.00% | OQ422566 |
| 86 | *Fusarium sambucinum* SC | *Fusarium graminearum* | LT222056 | 100.00% | OQ422567 |
| 87 | *Fusarium sambucinum* SC | *Fusarium graminearum* | MT185460 | 100.00% | OQ422568 |
| 88 | *Fusarium sambucinum* SC | *Fusarium graminearum* | MG274308 | 99.82% | OQ422569 |
| 89 | *Fusarium sambucinum* SC | *Fusarium graminearum* | KR047057 | 100.00% | OQ422570 |
| 90 | *Fusarium sambucinum* SC | *Fusarium graminearum* | ON054308 | 100.00% | OQ422571 |
| 91 | *Fusarium sambucinum* SC | *Fusarium graminearum* | OL364745 | 99.47% | OQ422572 |
| 92 | *Fusarium sambucinum* SC | *Fusarium graminearum* | MG274308 | 100.00% | OQ422491 |
| 93 | *Fusarium sambucinum* SC | *Fusarium graminearum* | KY910862 | 99.82% | OQ422492 |
| 94 | *Fusarium sambucinum* SC | *Fusarium graminearum* | HQ832817 | 100.00% | OQ422493 |
| 95 | *Fusarium sambucinum* SC | *Fusarium graminearum* | MG274308 | 100.00% | OQ422494 |
| 96 | *Fusarium sambucinum* SC | *Fusarium graminearum* | ON054308 | 100.00% | OQ422495 |
| 97 | *Fusarium sambucinum* SC | *Fusarium graminearum* | MW53472 | 99.80% | OQ422496 |
| 98 | *Fusarium sambucinum* SC | *Fusarium graminearum* | KY910868 | 100.00% | OQ422497 |
| 99 | *Fusarium sambucinum* SC | *Fusarium graminearum* | LT222056 | 100.00% | OQ422498 |
| 100 | *Fusarium sambucinum* SC | *Fusarium graminearum* | LT222056 | 100.00% | OQ422499 |
| 101 | *Fusarium sambucinum* SC | *Fusarium graminearum* | LT222056 | 99.65% | OQ422500 |
| 102 | *Fusarium sambucinum* SC | *Fusarium graminearum* | MK630104 | 100.00% | OQ422501 |
| 103 | *Fusarium sambucinum* SC | *Fusarium graminearum* | KY466825 | 100.00% | OQ422502 |
| 104 | *Fusarium sambucinum* SC | *Fusarium graminearum* | MT185460 | 100.00% | OQ422503 |
| 105 | *Fusarium sambucinum* SC | *Fusarium graminearum* | KY910868 | 99.46% | OQ422504 |
| 106 | *Fusarium sambucinum* SC | *Fusarium graminearum* | LT222056 | 100.00% | OQ422505 |
| 107 | *Fusarium sambucinum* SC | *Fusarium graminearum* | MG274308 | 100.00% | OQ422506 |
| 108 | *Fusarium sambucinum* SC | *Fusarium graminearum* | KR047057 | 99.83% | OQ422507 |
| 109 | *Fusarium sambucinum* SC | *Fusarium graminearum* | MH299910 | 100.00% | OQ422508 |
| 110 | *Fusarium sambucinum* SC | *Fusarium graminearum* | LT222056 | 99.82% | OQ422509 |
| 111 | *Fusarium sambucinum* SC | *Fusarium graminearum* | MG274308 | 100.00% | OQ422510 |
| 112 | *Fusarium sambucinum* SC | *Fusarium graminearum* | MG274308 | 100.00% | OQ422511 |
| 113 | *Fusarium sambucinum* SC | *Fusarium graminearum* | MG274308 | 100.00% | OQ422512 |
| 114 | *Fusarium sambucinum* SC | *Fusarium graminearum* | MG274308 | 100.00% | OQ422513 |
| 115 | *Fusarium sambucinum* SC | *Fusarium graminearum* | OL364745 | 100.00% | OQ422514 |
| 116 | *Fusarium sambucinum* SC | *Fusarium graminearum* | LT222056 | 99.82% | OQ422515 |
| 117 | *Fusarium sambucinum* SC | *Fusarium graminearum* | MH108136 | 99.82% | OQ422516 |
| 118 | *Fusarium sambucinum* SC | *Fusarium graminearum* | MG274308 | 100.00% | OQ422517 |
| 119 | *Fusarium sambucinum* SC | *Fusarium graminearum* | MT185456 | 99.82% | OQ422518 |
| 120 | *Fusarium sambucinum* SC | *Fusarium graminearum* | MK460853 | 99.64% | OQ422519 |
| 121 | *Fusarium sambucinum* SC | *Fusarium graminearum* | MK460853 | 100.00% | OQ422520 |
| 122 | *Fusarium sambucinum* SC | *Fusarium graminearum* | LT222056 | 99.65% | OQ422521 |
| 123 | *Fusarium sambucinum* SC | *Fusarium graminearum* | KY910868 | 100.00% | OQ422522 |
| 124 | *Fusarium sambucinum* SC | *Fusarium graminearum* | LT222056 | 100.00% | OQ422523 |
| 125 | *Fusarium sambucinum* SC | *Fusarium graminearum* | MG274308 | 100.00% | OQ422524 |
| 126 | *Fusarium sambucinum* SC | *Fusarium graminearum* | ON054308 | 99.82% | OQ422525 |
| 127 | *Fusarium sambucinum* SC | *Fusarium graminearum* | KY426427 | 99.60% | OQ422526 |
| 128 | *Fusarium sambucinum* SC | *Fusarium graminearum* | HQ832817 | 100.00% | OQ422527 |
| 129 | *Fusarium sambucinum* SC | *Fusarium graminearum* | OL364745 | 100.00% | OQ422528 |
| 130 | *Fusarium sambucinum* SC | *Fusarium graminearum* | OL364745 | 99.82% | OQ422529 |
| 131 | *Fusarium sambucinum* SC | *Fusarium graminearum* | MG274308 | 100.00% | OQ422530 |
| 132 | *Fusarium sambucinum* SC | *Fusarium graminearum* | MT185460 | 100.00% | OQ422531 |
| 133 | *Fusarium sambucinum* SC | *Fusarium graminearum* | MG274308 | 100.00% | OQ422532 |
| 134 | *Fusarium sambucinum* SC | *Fusarium graminearum* | LT222056 | 99.65% | OQ422533 |
| 135 | *Fusarium sambucinum* SC | *Fusarium graminearum* | KR047057 | 99.83% | OQ422534 |
| 136 | *Fusarium sambucinum* SC | *Fusarium graminearum* | MT185460 | 100.00% | OQ422535 |
| 137 | *Fusarium sambucinum* SC | *Fusarium graminearum* | KY910868 | 100.00% | OQ422536 |
| 138 | *Fusarium sambucinum* SC | *Fusarium graminearum* | HQ832817 | 99.82% | OQ422537 |
| 139 | *Fusarium sambucinum* SC | *Fusarium graminearum* | MT185460 | 100.00% | OQ422538 |
| 140 | *Fusarium sambucinum* SC | *Fusarium graminearum* | KY272801 | 99.80% | OQ422539 |
| 141 | *Fusarium sambucinum* SC | *Fusarium graminearum* | OL364745 | 99.47% | OQ422540 |
| 142 | *Fusarium sambucinum* SC | *Fusarium graminearum* | KY910868 | 100.00% | OQ422541 |
| 143 | *Fusarium sambucinum* SC | *Fusarium graminearum* | MT185456 | 100.00% | OQ422542 |
| 144 | *Fusarium sambucinum* SC | *Fusarium graminearum* | MT185460 | 100.00% | OQ422543 |
| 145 | *Fusarium sambucinum* SC | *Fusarium graminearum* | MT185460 | 100.00% | OQ422544 |
| 146 | *Fusarium sambucinum* SC | *Fusarium graminearum* | LT222056 | 100.00% | OQ422545 |
| 147 | *Fusarium sambucinum* SC | *Fusarium graminearum* | LT222056 | 100.00% | OQ422546 |
| 148 | *Fusarium sambucinum* SC | *Fusarium graminearum* | KY466825 | 99.80% | OQ422547 |
| 149 | *Fusarium sambucinum* SC | *Fusarium graminearum* | LT222056 | 99.82% | OQ422548 |
| 150 | *Fusarium sambucinum* SC | *Fusarium graminearum* | KY466825 | 100.00% | OQ422549 |
| 151 | *Fusarium sambucinum* SC | *Fusarium graminearum* | KY272779 | 99.61% | OQ422550 |
| 152 | *Fusarium sambucinum* SC | *Fusarium graminearum* | LT222056 | 100.00% | OQ422551 |
| 153 | *Fusarium sambucinum* SC | *Fusarium graminearum* | ON054308 | 99.82% | OQ422552 |
| 154 | *Fusarium sambucinum* SC | *Fusarium graminearum* | MT185456 | 99.64% | OQ422553 |
| 155 | *Fusarium sambucinum* SC | *Fusarium graminearum* | MK079918 | 99.46% | OQ422554 |
| 156 | *Fusarium sambucinum* SC | *Fusarium graminearum* | CP087874 | 99.82% | OQ422555 |
| 157 | *Fusarium sambucinum* SC | *Fusarium graminearum* | MG274308 | 99.82% | OQ422556 |
| 158 | *Fusarium sambucinum* SC | *Fusarium graminearum* | HQ832817 | 100.00% | OQ422557 |
| 159 | *Fusarium sambucinum* SC | *Fusarium graminearum* | CP087874 | 100.00% | OQ422558 |
| 160 | *Fusarium sambucinum* SC | *Fusarium graminearum* | MT185460 | 100.00% | OQ422559 |
| 161 | *Fusarium sambucinum* SC | *Fusarium graminearum* | LT222056 | 100.00% | OQ422560 |
| 162 | *Fusarium sambucinum* SC | *Fusarium graminearum* | KR047057 | 99.80% | OQ422561 |
| 163 | *Fusarium sambucinum* SC | *Fusarium graminearum* | OL364745 | 99.47% | OQ422562 |
| 164 | *Fusarium sambucinum* SC | *Fusarium graminearum* | MG274308 | 99.47% | OQ422563 |
| 165 | *Fusarium sambucinum* SC | *Fusarium graminearum* | MT185460 | 100.00% | OQ422564 |
| 166 | *Fusarium* spp. | *Fusarium incarnatum* | [MN882828](https://www.ncbi.nlm.nih.gov/nucleotide/MN882828.1?report=genbank&log$=nucltop&blast_rank=1&RID=YGG2S1RP013) | 100.00% | OQ422640 |
| 167 | *Fusarium* spp. | *Fusarium incarnatum* | MN882828 | 99.83% | OQ422641 |
| 168 | *Fusarium* spp. | *Fusarium incarnatum* | [MN227262](https://www.ncbi.nlm.nih.gov/nucleotide/MN227262.1?report=genbank&log$=nucltop&blast_rank=27&RID=YGGDTAYG013) | 99.65% | OQ422642 |
| 169 | *Fusarium* spp. | *Fusarium incarnatum* | [MH979697](https://www.ncbi.nlm.nih.gov/nucleotide/MH979697.1?report=genbank&log$=nucltop&blast_rank=11&RID=YGGHVC94013) | 99.65% | OQ422643 |
| 170 | *Fusarium* spp. | *Fusarium incarnatum* | [MN882829](https://www.ncbi.nlm.nih.gov/nucleotide/MN882829.1?report=genbank&log$=nucltop&blast_rank=6&RID=YGGMC11X013) | 99.82% | OQ422644 |
| 171 | *Fusarium* spp. | *Fusarium incarnatum* | [MH865892](https://www.ncbi.nlm.nih.gov/nucleotide/MH865892.1?report=genbank&log$=nucltop&blast_rank=12&RID=YGGPDK6V016) | 100.00% | OQ422645 |
| 172 | *Fusarium* spp. | *Fusarium incarnatum* | [MN882828](https://www.ncbi.nlm.nih.gov/nucleotide/MN882828.1?report=genbank&log$=nucltop&blast_rank=1&RID=YGGTENGS013) | 100.00% | OQ422646 |
| 173 | *Fusarium* spp. | *Fusarium incarnatum* | [MN882828](https://www.ncbi.nlm.nih.gov/nucleotide/MN882828.1?report=genbank&log$=nucltop&blast_rank=6&RID=YGGVTYSF013) | 99.83% | OQ422647 |
| 174 | *Fusarium* spp. | *Fusarium incarnatum* | [MH290471](https://www.ncbi.nlm.nih.gov/nucleotide/MH290471.1?report=genbank&log$=nucltop&blast_rank=9&RID=YGGY232N013) | 99.46% | OQ422648 |
| 175 | *Fusarium* spp. | *Fusarium incarnatum* | [MN227262](https://www.ncbi.nlm.nih.gov/nucleotide/MN227262.1?report=genbank&log$=nucltop&blast_rank=1&RID=YGH16R88013) | 100.00% | OQ422649 |
| 176 | *Fusarium* spp. | *Fusarium incarnatum* | [MN882828](https://www.ncbi.nlm.nih.gov/nucleotide/MN882828.1?report=genbank&log$=nucltop&blast_rank=7&RID=YGH3VHGD016) | 99.82% | OQ422650 |
| 177 | *Fusarium* spp. | *Fusarium incarnatum* | [MH290471](https://www.ncbi.nlm.nih.gov/nucleotide/MH290471.1?report=genbank&log$=nucltop&blast_rank=2&RID=YGH77YSK016) | 99.82% | OQ422651 |
| 178 | *Fusarium* spp. | *Fusarium incarnatum* | [MH290471](https://www.ncbi.nlm.nih.gov/nucleotide/MH290471.1?report=genbank&log$=nucltop&blast_rank=2&RID=YGH9G2DB013) | 99.82% | OQ422652 |
| 179 | *Fusarium* spp. | *Fusarium incarnatum* | [MN882829](https://www.ncbi.nlm.nih.gov/nucleotide/MN882829.1?report=genbank&log$=nucltop&blast_rank=1&RID=YGHBPU37016) | 100.00% | OQ422653 |
| 180 | *Fusarium* spp. | *Fusarium incarnatum* | [MH290471](https://www.ncbi.nlm.nih.gov/nucleotide/MH290471.1?report=genbank&log$=nucltop&blast_rank=13&RID=YGHEVRPS013) | 99.64% | OQ422654 |
| 181 | *Fusarium* spp. | *Fusarium incarnatum* | [MN882828](https://www.ncbi.nlm.nih.gov/nucleotide/MN882828.1?report=genbank&log$=nucltop&blast_rank=1&RID=YGHHYET9013) | 100.00% | OQ422655 |
| 182 | *Fusarium* spp. | *Fusarium incarnatum* | [MH290471](https://www.ncbi.nlm.nih.gov/nucleotide/MH290471.1?report=genbank&log$=nucltop&blast_rank=2&RID=YGHM85EP013) | 99.64% | OQ422656 |
| 183 | *Fusarium* spp. | *Fusarium incarnatum* | MN882828 | 99.30% | OQ422657 |
| 184 | *Fusarium* spp. | *Fusarium incarnatum* | [MN882828](https://www.ncbi.nlm.nih.gov/nucleotide/MN882828.1?report=genbank&log$=nucltop&blast_rank=4&RID=YGHRG85201R) | 100.00% | OQ422658 |
| 185 | *Fusarium* spp. | *Fusarium incarnatum* | [MW958219](https://www.ncbi.nlm.nih.gov/nucleotide/MW958219.1?report=genbank&log$=nucltop&blast_rank=18&RID=YGHTMMHC01R) | 99.64% | OQ422659 |
| 186 | *Fusarium* spp. | *Fusarium incarnatum* | [MN882829](https://www.ncbi.nlm.nih.gov/nucleotide/MN882829.1?report=genbank&log$=nucltop&blast_rank=2&RID=YGHYCNMW01R) | 99.82% | OQ422660 |
| 187 | *Fusarium* spp. | *Fusarium incarnatum* | [MN882828](https://www.ncbi.nlm.nih.gov/nucleotide/MN882828.1?report=genbank&log$=nucltop&blast_rank=9&RID=YGJ0UJ9R013) | 99.82% | OQ422661 |
| 188 | *Fusarium* spp. | *Fusarium incarnatum* | [MN227262](https://www.ncbi.nlm.nih.gov/nucleotide/MN227262.1?report=genbank&log$=nucltop&blast_rank=1&RID=YGJ3C6K1013) | 100.00% | OQ422662 |
| 189 | *Fusarium* spp. | *Fusarium incarnatum* | [MN882828](https://www.ncbi.nlm.nih.gov/nucleotide/MN882828.1?report=genbank&log$=nucltop&blast_rank=1&RID=YGJ53GA2013) | 100.00% | OQ422663 |
| 190 | *Fusarium* spp. | *Fusarium incarnatum* | [MN882828](https://www.ncbi.nlm.nih.gov/nucleotide/MN882828.1?report=genbank&log$=nucltop&blast_rank=8&RID=YGJ8HCV7013) | 99.83% | OQ422664 |
| 191 | *Fusarium* spp. | *Fusarium incarnatum* | [MH290471](https://www.ncbi.nlm.nih.gov/nucleotide/MH290471.1?report=genbank&log$=nucltop&blast_rank=5&RID=YGJAS8SF013) | 99.82% | OQ422665 |
| 192 | *Fusarium* spp. | *Fusarium incarnatum* | [MH290470](https://www.ncbi.nlm.nih.gov/nucleotide/MH290470.1?report=genbank&log$=nucltop&blast_rank=5&RID=YGJCR48G013) | 99.82% | OQ422666 |
| 193 | *Fusarium* spp. | *Fusarium incarnatum* | [MH290471](https://www.ncbi.nlm.nih.gov/nucleotide/MH290471.1?report=genbank&log$=nucltop&blast_rank=11&RID=YGJEJ403013) | 99.64% | OQ422667 |
| 194 | *Fusarium* spp. | *Fusarium incarnatum* | MN882828 | 99.47% | OQ422668 |
| 195 | *Fusarium* spp. | *Fusarium incarnatum* | [MH290470](https://www.ncbi.nlm.nih.gov/nucleotide/MH290470.1?report=genbank&log$=nucltop&blast_rank=1&RID=YGJXKCUU013) | 99.82% | OQ422669 |
| 196 | *Fusarium* spp. | *Fusarium incarnatum* | [MN882828](https://www.ncbi.nlm.nih.gov/nucleotide/MN882828.1?report=genbank&log$=nucltop&blast_rank=3&RID=YGJZFK19013) | 100.00% | OQ422670 |
| 197 | *Fusarium* spp. | *Fusarium incarnatum* | [MN882828](https://www.ncbi.nlm.nih.gov/nucleotide/MN882828.1?report=genbank&log$=nucltop&blast_rank=1&RID=YGK1CPC9013) | 100.00% | OQ422671 |
| 198 | *Fusarium* spp. | *Fusarium incarnatum* | [MN882829](https://www.ncbi.nlm.nih.gov/nucleotide/MN882829.1?report=genbank&log$=nucltop&blast_rank=2&RID=YGK34DG9013) | 99.82% | OQ422672 |
| 199 | *Fusarium* spp. | *Fusarium incarnatum* | [MH290470](https://www.ncbi.nlm.nih.gov/nucleotide/MH290470.1?report=genbank&log$=nucltop&blast_rank=1&RID=YGK4NXH9016) | 99.82% | OQ422673 |
| 200 | *Fusarium* spp. | *Fusarium incarnatum* | [MN882828](https://www.ncbi.nlm.nih.gov/nucleotide/MN882828.1?report=genbank&log$=nucltop&blast_rank=1&RID=YGK6P8W6013) | 100.00% | OQ422674 |
| 201 | *Fusarium* spp. | *Fusarium incarnatum* | MH865892 | 99.63% | OQ422675 |
| 202 | *Fusarium* spp. | *Fusarium incarnatum* | [MN882828](https://www.ncbi.nlm.nih.gov/nucleotide/MN882828.1?report=genbank&log$=nucltop&blast_rank=4&RID=YGKC0ZNR013) | 100.00% | OQ422676 |
| 203 | *Fusarium* spp. | *Fusarium incarnatum* | [MN227262](https://www.ncbi.nlm.nih.gov/nucleotide/MN227262.1?report=genbank&log$=nucltop&blast_rank=1&RID=YGKEDB7S013) | 99.82% | OQ422677 |
| 204 | *Fusarium* spp. | *Fusarium incarnatum* | [MT565585](https://www.ncbi.nlm.nih.gov/nucleotide/MT565585.1?report=genbank&log$=nucltop&blast_rank=4&RID=YGKG4RJF016) | 100.00% | OQ422678 |
| 205 | *Fusarium* spp. | *Fusarium incarnatum* | [MH290471](https://www.ncbi.nlm.nih.gov/nucleotide/MH290471.1?report=genbank&log$=nucltop&blast_rank=3&RID=YGKJDB5E013) | 99.82% | OQ422679 |
| 206 | *Fusarium* spp. | *Fusarium incarnatum* | [MN882828](https://www.ncbi.nlm.nih.gov/nucleotide/MN882828.1?report=genbank&log$=nucltop&blast_rank=9&RID=YGKM6ERZ016) | 99.83% | OQ422680 |
| 207 | *Fusarium* spp. | *Fusarium incarnatum* | [MH290470](https://www.ncbi.nlm.nih.gov/nucleotide/MH290470.1?report=genbank&log$=nucltop&blast_rank=22&RID=YGKP14WU013) | 99.47% | OQ422681 |
| 208 | *Fusarium* spp. | *Fusarium incarnatum* | [FN597588](https://www.ncbi.nlm.nih.gov/nucleotide/FN597588.1?report=genbank&log$=nucltop&blast_rank=14&RID=YGKSH0YY013) | 100.00% | OQ422682 |
| 209 | *Fusarium* spp. | *Fusarium incarnatum* | [MN882828](https://www.ncbi.nlm.nih.gov/nucleotide/MN882828.1?report=genbank&log$=nucltop&blast_rank=9&RID=YGKW93HA016) | 99.83% | OQ422683 |
| 210 | *Fusarium* spp. | *Fusarium incarnatum* | ON391483 | 99.38% | OQ422684 |
| 211 | *Fusarium* spp. | *Fusarium incarnatum* | [MH979697](https://www.ncbi.nlm.nih.gov/nucleotide/MH979697.1?report=genbank&log$=nucltop&blast_rank=1&RID=YGKYSU9T013) | 99.82% | OQ422685 |
| 212 | *Fusarium* spp. | *Fusarium incarnatum* | MN227262 | 99.30% | OQ422686 |
| 213 | *Fusarium* spp. | *Fusarium incarnatum* | [MH290470](https://www.ncbi.nlm.nih.gov/nucleotide/MH290470.1?report=genbank&log$=nucltop&blast_rank=2&RID=YGM0MREK013) | 99.64% | OQ422687 |
| 214 | *Fusarium* spp. | *Fusarium incarnatum* | [MN882828](https://www.ncbi.nlm.nih.gov/nucleotide/MN882828.1?report=genbank&log$=nucltop&blast_rank=3&RID=YGM2NW33013) | 100.00% | OQ422688 |
| 215 | *Fusarium* spp. | *Fusarium incarnatum* | MN227262 | 99.65% | OQ422689 |
| 216 | *Fusarium* spp. | *Fusarium incarnatum* | [MW958219](https://www.ncbi.nlm.nih.gov/nucleotide/MW958219.1?report=genbank&log$=nucltop&blast_rank=13&RID=YGM4EGA2013) | 99.64% | OQ422690 |
| 217 | *Fusarium* spp. | *Fusarium incarnatum* | [MH979697](https://www.ncbi.nlm.nih.gov/nucleotide/MH979697.1?report=genbank&log$=nucltop&blast_rank=1&RID=YGM6HN71013) | 99.83% | OQ422691 |
| 218 | *Fusarium* spp. | *Fusarium incarnatum* | [MW958219](https://www.ncbi.nlm.nih.gov/nucleotide/MW958219.1?report=genbank&log$=nucltop&blast_rank=7&RID=YGM8NABK01R) | 99.64% | OQ422692 |
| 219 | *Fusarium* spp. | *Fusarium incarnatum* | [MN882828](https://www.ncbi.nlm.nih.gov/nucleotide/MN882828.1?report=genbank&log$=nucltop&blast_rank=9&RID=YGMAP7XT01R) | 99.83% | OQ422693 |
| 220 | *Fusarium* spp. | *Fusarium incarnatum* | [MW958219](https://www.ncbi.nlm.nih.gov/nucleotide/MW958219.1?report=genbank&log$=nucltop&blast_rank=3&RID=YGMDJETH01R) | 100.00% | OQ422694 |
| 221 | *Fusarium* spp. | *Fusarium incarnatum* | [MH979697](https://www.ncbi.nlm.nih.gov/nucleotide/MH979697.1?report=genbank&log$=nucltop&blast_rank=13&RID=YGMFNUNV01R) | 99.64% | OQ422695 |
| 222 | *Fusarium* spp. | *Fusarium incarnatum* | [MN882828](https://www.ncbi.nlm.nih.gov/nucleotide/MN882828.1?report=genbank&log$=nucltop&blast_rank=3&RID=YGMHFDWF01R) | 100.00% | OQ422696 |
| 223 | *Fusarium* spp. | *Fusarium incarnatum* | [MN882828](https://www.ncbi.nlm.nih.gov/nucleotide/MN882828.1?report=genbank&log$=nucltop&blast_rank=3&RID=YGMM3UY101R) | 100.00% | OQ422697 |
| 224 | *Fusarium* spp. | *Fusarium incarnatum* | [MN882828](https://www.ncbi.nlm.nih.gov/nucleotide/MN882828.1?report=genbank&log$=nucltop&blast_rank=9&RID=YGMPDZ6H01R) | 99.82% | OQ422698 |
| 225 | *Fusarium* spp. | *Fusarium incarnatum* | [MH290471](https://www.ncbi.nlm.nih.gov/nucleotide/MH290471.1?report=genbank&log$=nucltop&blast_rank=2&RID=YGMSAHTA01R) | 99.82% | OQ422699 |
| 226 | *Fusarium* spp. | *Fusarium incarnatum* | [MN882829](https://www.ncbi.nlm.nih.gov/nucleotide/MN882829.1?report=genbank&log$=nucltop&blast_rank=39&RID=YGMU888P01R) | 99.65% | OQ422700 |
| 227 | *Fusarium* spp. | *Fusarium incarnatum* | [MN882828](https://www.ncbi.nlm.nih.gov/nucleotide/MN882828.1?report=genbank&log$=nucltop&blast_rank=9&RID=YGMXFZJ4013) | 99.83% | OQ422701 |
| 228 | *Fusarium* spp. | *Fusarium incarnatum* | [MN882828](https://www.ncbi.nlm.nih.gov/nucleotide/MN882828.1?report=genbank&log$=nucltop&blast_rank=4&RID=YGMZS82Z016) | 100.00% | OQ422702 |
| 229 | *Fusarium* spp. | *Fusarium incarnatum* | MH865892 | 100.00% | OQ422703 |
| 230 | *Fusarium* spp. | *Fusarium incarnatum* | [MN882828](https://www.ncbi.nlm.nih.gov/nucleotide/MN882828.1?report=genbank&log$=nucltop&blast_rank=9&RID=YGN1E62S013) | 99.83% | OQ422704 |
| 231 | *Fusarium* spp. | *Fusarium incarnatum* | [MN882828](https://www.ncbi.nlm.nih.gov/nucleotide/MN882828.1?report=genbank&log$=nucltop&blast_rank=4&RID=YGN4147X013) | 100.00% | OQ422705 |
| 232 | *Fusarium* spp. | *Fusarium incarnatum* | [ON391483](https://www.ncbi.nlm.nih.gov/nucleotide/ON391483.1?report=genbank&log$=nucltop&blast_rank=2&RID=YGN60HX8013) | 100.00% | OQ422706 |
| 233 | *Fusarium* spp. | *Fusarium equiseti* | MT558602 | 99.82% | OQ421745 |
| 234 | *Fusarium* spp. | *Fusarium equiseti* | JQ936180 | 100.00% | OQ421746 |
| 235 | *Fusarium* spp. | *Fusarium equiseti* | KY523100 | 99.83% | OQ421747 |
| 236 | *Fusarium* spp. | *Fusarium equiseti* | JQ936153 | 99.82% | OQ421748 |
| 237 | *Fusarium* spp. | *Fusarium equiseti* | MT558570 | 100.00% | OQ421749 |
| 238 | *Fusarium* spp. | *Fusarium equiseti* | OQ248231 | 99.83% | OQ421750 |
| 239 | *Fusarium* spp. | *Fusarium equiseti* | MH054915 | 100.00% | OQ421751 |
| 240 | *Fusarium* spp. | *Fusarium equiseti* | OM327434 | 100.00% | OQ421752 |
| 241 | *Fusarium* spp. | *Fusarium equiseti* | MH879250 | 100.00% | OQ421753 |
| 242 | *Fusarium* spp. | *Fusarium equiseti* | MH578585 | 100.00% | OQ421754 |
| 243 | *Fusarium* spp. | *Fusarium equiseti* | MH578585 | 100.00% | OQ421755 |
| 244 | *Fusarium* spp. | *Fusarium equiseti* | KX576658 | 99.64% | OQ421756 |
| 245 | *Fusarium* spp. | *Fusarium equiseti* | MT558602 | 99.82% | OQ422854 |
| 246 | *Fusarium* spp. | *Fusarium equiseti* | OQ031179 | 99.47% | OQ422855 |
| 247 | *Fusarium* spp. | *Fusarium equiseti* | OQ248231 | 100.00% | OQ421757 |
| 248 | *Fusarium* spp. | *Fusarium equiseti* | MW497628 | 100.00% | OQ421758 |
| 249 | *Fusarium* spp. | *Fusarium equiseti* | MT428185 | 100.00% | OQ421759 |
| 250 | *Fusarium* spp. | *Fusarium equiseti* | MK334366 | 100.00% | OQ421760 |
| 251 | *Fusarium* spp. | *Fusarium equiseti* | KX463031 | 99.80% | OQ421761 |
| 252 | *Fusarium* spp. | *Fusarium equiseti* | KT277307 | 100.00% | OQ421762 |
| 253 | *Fusarium* spp. | *Fusarium equiseti* | MH707073 | 99.81% | OQ421763 |
| 254 | *Fusarium* spp. | *Fusarium equiseti* | KR364597 | 100.00% | OQ421764 |
| 255 | *Fusarium* spp. | *Fusarium equiseti* | EU326202 | 99.82% | OQ421765 |
| 256 | *Fusarium* spp. | *Fusarium equiseti* | OQ248231 | 99.83% | OQ421766 |
| 257 | *Fusarium* spp. | *Fusarium equiseti* | KT277307 | 100.00% | OQ421767 |
| 258 | *Fusarium* spp. | *Fusarium equiseti* | OM956012 | 100.00% | OQ421768 |
| 259 | *Fusarium* spp. | *Fusarium equiseti* | KT277307 | 100.00% | OQ421769 |
| 260 | *Fusarium* spp. | *Fusarium equiseti* | LC514690 | 99.79% | OQ421770 |
| 261 | *Fusarium* spp. | *Fusarium equiseti* | MK334366 | 100.00% | OQ421771 |
| 262 | *Fusarium* spp. | *Fusarium equiseti* | MT428185 | 100.00% | OQ421772 |
| 263 | *Fusarium* spp. | *Fusarium equiseti* | MH054915 | 100.00% | OQ421773 |
| 264 | *Fusarium* spp. | *Fusarium equiseti* | MH578585 | 100.00% | OQ421774 |
| 265 | *Fusarium sambucinum* SC | *Fusarium boothii* | MK910046 | 99.80% | OQ421787 |
| 266 | *Fusarium sambucinum* SC | *Fusarium boothii* | MK910046 | 99.60% | OQ421788 |
| 267 | *Fusarium sambucinum* SC | *Fusarium boothii* | MK910046 | 99.60% | OQ421789 |
| 268 | *Fusarium sambucinum* SC | *Fusarium boothii* | NR121203 | 99.79% | OQ421790 |
| 269 | *Fusarium sambucinum* SC | *Fusarium boothii* | MK910046 | 99.40% | OQ421791 |
| 270 | *Fusarium sambucinum* SC | *Fusarium boothii* | KY466819 | 100.00% | OQ421792 |
| 271 | *Fusarium sambucinum* SC | *Fusarium boothii* | NR121203 | 99.82% | OQ422856 |
| 272 | *Fusarium sambucinum* SC | *Fusarium boothii* | MK910046 | 99.80% | OQ421793 |
| 273 | *Fusarium sambucinum* SC | *Fusarium boothii* | MK910046 | 99.60% | OQ421794 |
| 274 | *Fusarium sambucinum* SC | *Fusarium boothii* | KY767623 | 99.38% | OQ422857 |
| 275 | *Fusarium sambucinum* SC | *Fusarium boothii* | NR121203 | 100.00% | OQ422858 |
| 276 | *Fusarium sambucinum* SC | *Fusarium boothii* | KY767623 | 99.40% | OQ422859 |
| 277 | *Fusarium sambucinum* SC | *Fusarium boothii* | MK756046 | 99.40% | OQ421795 |
| 278 | *Fusarium sambucinum* SC | *Fusarium culmorum* | MF372583 | 99.63% | OQ421775 |
| 279 | *Fusarium sambucinum* SC | *Fusarium culmorum* | MK910046 | 100% | OQ422860 |
| 280 | *Fusarium sambucinum* SC | *Fusarium culmorum* | MH681148 | 100.00% | OQ421776 |
| 281 | *Fusarium sambucinum* SC | *Fusarium culmorum* | MH260597 | 99.80% | OQ421777 |
| 282 | *Fusarium sambucinum* SC | *Fusarium culmorum* | MZ089678 | 99.80% | OQ421778 |
| 283 | *Fusarium sambucinum* SC | *Fusarium culmorum* | KC329615 | 99.59% | OQ421779 |
| 284 | *Fusarium sambucinum* SC | *Fusarium culmorum* | MF372583 | 100.00% | OQ421780 |
| 285 | *Fusarium sambucinum* SC | *Fusarium culmorum* | KC329615 | 99.80% | OQ421781 |
| 286 | *Fusarium sambucinum* SC | *Fusarium culmorum* | MZ089678 | 99.80% | OQ421782 |
| 287 | *Fusarium sambucinum* SC | *Fusarium culmorum* | MZ089684 | 100.00% | OQ421783 |
| 288 | *Fusarium sambucinum* SC | *Fusarium culmorum* | MH282513 | 99.80% | OQ421784 |
| 289 | *Fusarium fujikuroi* SC | *Fusarium proliferatum* | KJ767073 | 100.00% | OQ421596 |
| 290 | *Fusarium fujikuroi* SC | *Fusarium proliferatum* | MT466521 | 100.00% | OQ421597 |
| 291 | *Fusarium fujikuroi* SC | *Fusarium proliferatum* | MK814399 | 99.83% | OQ421598 |
| 292 | *Fusarium fujikuroi* SC | *Fusarium proliferatum* | MG562501 | 100.00% | OQ421599 |
| 293 | *Fusarium fujikuroi* SC | *Fusarium proliferatum* | MW308366 | 100.00% | OQ421600 |
| 294 | *Fusarium fujikuroi* SC | *Fusarium verticillioides* | OP237229 | 99.82% | OQ421601 |
| 295 | *Fusarium fujikuroi* SC | *Fusarium verticillioides* | OP237229 | 99.82% | OQ421602 |
| 296 | *Fusarium* spp. | *Fusarium chlamydosporum* | HF570009 | 99.80% | OQ421603 |
| 297 | *Aspergillus* section *flavi* | *Aspergillus flavus* | LC602027 | 99.51% | OQ422920 |
| 298 | *Aspergillus* section *flavi* | *Aspergillus flavus* | MK450361 | 99.83% | OQ422921 |
| 299 | *Aspergillus* section *flavi* | *Aspergillus flavus* | OP596223 | 100.00% | OQ422922 |
| 300 | *Aspergillus* section *flavi* | *Aspergillus flavus* | MT645322 | 100.00% | OQ422923 |
| 301 | *Aspergillus* section *flavi* | *Aspergillus flavus* | LC105697 | 100.00% | OQ422924 |
| 302 | *Aspergillus* section *flavi* | *Aspergillus flavus* | MT645322 | 100.00% | OQ422925 |
| 303 | *Aspergillus* section *flavi* | *Aspergillus flavus* | MK450361 | 99.83% | OQ422926 |
| 304 | *Aspergillus* section *flavi* | *Aspergillus flavus* | MT645322 | 100.00% | OQ422927 |
| 305 | *Aspergillus* section *flavi* | *Aspergillus flavus* | OP596226 | 100.00% | OQ422928 |
| 306 | *Aspergillus* section *flavi* | *Aspergillus flavus* | CP051065 | 100.00% | OQ422929 |
| 307 | *Aspergillus* section *flavi* | *Aspergillus flavus* | LC602027 | 100.00% | OQ422930 |
| 308 | *Aspergillus* section *flavi* | *Aspergillus flavus* | MW600444 | 100.00% | OQ422931 |
| 309 | *Aspergillus* section *flavi* | *Aspergillus flavus* | MN559662 | 100.00% | OQ422932 |
| 310 | *Aspergillus* section *flavi* | *Aspergillus flavus* | MN180857 | 100.00% | OQ422933 |
| 311 | *Aspergillus* section *flavi* | *Aspergillus flavus* | MN180857 | 100.00% | OQ422934 |
| 312 | *Aspergillus* section *flavi* | *Aspergillus flavus* | MN180857 | 100.00% | OQ422935 |
| 313 | *Aspergillus* section *flavi* | *Aspergillus flavus* | KX067853 | 100.00% | OQ422936 |
| 314 | *Aspergillus* section *flavi* | *Aspergillus flavus* | MT447484 | 100.00% | OQ422937 |
| 315 | *Aspergillus* section *flavi* | *Aspergillus flavus* | MN095143 | 100.00% | OQ422938 |
| 316 | *Aspergillus* section *flavi* | *Aspergillus flavus* | MK450361 | 100.00% | OQ422939 |
| 317 | *Aspergillus* section *flavi* | *Aspergillus flavus* | MT420620 | 100.00% | OQ422940 |
| 318 | *Aspergillus* section *flavi* | *Aspergillus flavus* | MN180857 | 100.00% | OQ422941 |
| 319 | *Aspergillus* section *flavi* | *Aspergillus flavus* | MN095124 | 99.66% | OQ422942 |
| 320 | *Aspergillus* section *flavi* | *Aspergillus flavus* | LC602022 | 100.00% | OQ422943 |
| 321 | *Aspergillus* section *flavi* | *Aspergillus flavus* | MN180857 | 99.49% | OQ422944 |
| 322 | *Aspergillus* section *flavi* | *Aspergillus flavus* | MN180857 | 100.00% | OQ422945 |
| 323 | *Aspergillus* section *flavi* | *Aspergillus flavus* | MT645322 | 100.00% | OQ422946 |
| 324 | *Aspergillus* section *flavi* | *Aspergillus flavus* | MN856403 | 99.83% | OQ422947 |
| 325 | *Aspergillus* section *flavi* | *Aspergillus flavus* | KX067853 | 100.00% | OQ422948 |
| 326 | *Aspergillus fumigatus* | *Aspergillus fumigatus* | OW984496 | 99.67% | OQ421619 |
| 327 | *Aspergillus fumigatus* | *Aspergillus fumigatus* | MK968249 | 100.00% | OQ421620 |
| 328 | *Aspergillus fumigatus* | *Aspergillus fumigatus* | JN851039 | 100.00% | OQ421621 |
| 329 | *Aspergillus fumigatus* | *Aspergillus fumigatus* | MK623263 | 99.67% | OQ422949 |
| 330 | *Aspergillus fumigatus* | *Aspergillus fumigatus* | MT558940 | 100.00% | OQ421622 |
| 331 | *Aspergillus fumigatus* | *Aspergillus fumigatus* | MK817529 | 100.00% | OQ421623 |
| 332 | *Aspergillus fumigatus* | *Aspergillus fumigatus* | MK968249 | 99.63% | OQ422950 |
| 333 | *Aspergillus fumigatus* | *Aspergillus fumigatus* | MT558940 | 100.00% | OQ421624 |
| 334 | *Aspergillus fumigatus* | *Aspergillus fumigatus* | JN851039 | 100.00% | OQ421625 |
| 335 | *Aspergillus fumigatus* | *Aspergillus fumigatus* | MK623263 | 100.00% | OQ421626 |
| 336 | *Aspergillus fumigatus* | *Aspergillus fumigatus* | KU687812 | 100.00% | OQ421627 |
| 337 | *Aspergillus fumigatus* | *Aspergillus fumigatus* | KU687812 | 100.00% | OQ421628 |
| 338 | *Aspergillus* spp. | *Aspergillus pseudoglaucus* | MH137909 | 100.00% | OQ421635 |
| 339 | *Aspergillus* spp. | *Aspergillus pseudoglaucus* | MT316341 | 100.00% | OQ421636 |
| 340 | *Aspergillus* spp. | *Aspergillus pseudoglaucus* | MH137923 | 100.00% | OQ421637 |
| 341 | *Aspergillus* spp. | *Aspergillus pseudoglaucus* | MT316341 | 100.00% | OQ421638 |
| 342 | *Aspergillus* spp. | *Aspergillus pseudoglaucus* | OW988438 | 99.63% | OQ421639 |
| 343 | *Aspergillus* spp. | *Aspergillus ruber* | KU686683 | 100.00% | OQ421640 |
| 344 | *Aspergillus* spp. | *Aspergillus sclerotiorum* | OL772720 | 99.22% | OQ421641 |
| 345 | *Aspergillus* spp. | *Aspergillus sclerotiorum* | OW987531 | 100.00% | OQ421642 |
| 346 | *Aspergillus* spp. | *Aspergillus sclerotiorum* | OW987531 | 100.00% | OQ421643 |
| 347 | *Aspergillus* spp. | *Aspergillus tamarii* | MN202674 | 100.00% | OQ421644 |
| 348 | *Aspergillus* spp. | *Aspergillus versicolor* | OP103945 | 100.00% | OQ421645 |
| 349 | *Aspergillus* spp. | *Aspergillus welwitschiae* | MK450668 | 100.00% | OQ421646 |

**Supplementary Table 2**. MS/MS parameters for the determination of 15 mycotoxins.

| **Mycotoxins** | **Dwell Time (s)** | **Precursor Ion (*m/z*)** | **Production**  **(*m/z*)** | **Cone Voltage (V)** | **Collision Energy (eV)** |
| --- | --- | --- | --- | --- | --- |
| AFB_1_ | 3.33 | 313.24 | 241.16*/284.97 | 74 | 36/22 |
| AFB_2_ | 3.17 | 315.22 | 259.12*/287.14 | 2 | 28/26 |
| AFG_1_ | 3.19 | 329.22 | 243.08*/199.86 | 12 | 26/42 |
| AFG_2_ | 3.04 | 331.22 | 189.05*/245.09 | 22 | 40/30 |
| DON | 1.99 | 297.10 | 249.10*/231.10 | 30 | 10/13 |
| ZEN | 4.21 | 317.24 | 175.05*/131.04 | 56 | 24/26 |
| FB_1_ | 2.97 | 722.61 | 334.33*/352.34 | 20 | 38/36 |
| FB_2_ | 3.43 | 706.81 | 336.50*/318.48 | 30 | 36/42 |
| FB_3_ | 3.24 | 706.81 | 336.50*/318.48 | 30 | 36/41 |
| AOH | 3.40 | 259.03 | 185.12*/213.13 | 64 | 28/24 |
| AME | 4.20 | 273.04 | 258.00*/128.06 | 54 | 25/26 |
| TeA | 1.69 | 198.10 | 125.02*/153.11 | 42 | 16/12 |
| TEN | 3.43 | 415.45 | 132.13*/256.23 | 14 | 34/34 |
| ALT | 3.04 | 292.97 | 257.07*/275.07 | 4 | 14/8 |
| ALS | 3.22 | 289.00 | 245.00*/230.10 | 45 | 17/20 |

Note: * Primary product ions.
